# Reply to Moline et al. Comment on “Alliance for Sleep Clinical Practice Guideline on Switching or Deprescribing Hypnotic Medications for Insomnia. *J. Clin. Med.* 2023, *12*, 2493”

**DOI:** 10.3390/jcm14051635

**Published:** 2025-02-28

**Authors:** Nathaniel F. Watson, Ruth M. Benca, Andrew D. Krystal, William V. McCall, David N. Neubauer

**Affiliations:** 1 Department of Neurology, University of Washington School of Medicine, Seattle, WA 98195, USA; 2 Department of Psychiatry and Behavioral Medicine, Wake Forest School of Medicine, Winston-Salem, NC 27101, USA; rbenca@wakehealth.edu; 3 Department of Psychiatry and Neurology, UCSF Weill Institute for Neurosciences, San Francisco, CA 94158, USA; andrew.krystal@ucsf.edu; 4 Department of Psychiatry and Health Behavior, Medical College of Georgia, Augusta University, Augusta, GA 30912, USA; wmccall@augusta.edu; 5 Department of Psychiatry and Behavioral Sciences, School of Medicine, Johns Hopkins University, Baltimore, MD 21218, USA; neubauer@jhmi.edu

We thank Moline et al. [1] for their interest in our manuscript, “Alliance for Sleep Clinical Practice Guideline on Switching or Deprescribing Hypnotic Medications for Insomnia.” [2]. To our knowledge, this is the first evidence-based clinical guideline to address the issue of deprescribing or switching medications in individuals with insomnia. We would like to respond to the concerns raised in the commentary by Moline and colleagues.

First, Moline et al. state, “There is a misinterpretation of results from a clinical study of lemborexant (E2006-G000-303, NCT02952820, SUNRISE 2) that appears in Table 1 of the aforementioned manuscript.” [3]. However, this study is not referenced in our manuscript, and as such we are confused by this comment. We reference four studies related to Lemborexant in our work, with the Takaesu et al. study being the primary resource for lemborexant in Table 1 [4].

Moline et al. assert that we were “conflating” one time point in one group of participants with the results for all participants. They also take issue with our statement, “*Those on lemborexant medication for 1 year stayed statistically significantly better than baseline for 2 weeks post-discontinuation and were significantly worse than at the end of double-blind treatment for two weeks after discontinuation for self-reported sleep onset latency (SOL).*” We agree that not all panels in Figure 2 tell the same story, and visually showing whether confidence intervals overlap is challenging [4]. However, our assessment of this Figure concludes that subjective sleep onset latency (sSOL) was worse following lemborexant discontinuation after 1–7 and 8–14 nights of follow up for LEM5-LEM5 and after 8–14 nights of follow up for PBO-LEM10 (both Figure 2, Panel a). This evidence supports our statements and conclusions.

Regarding subjective wake after sleep onset (sWASO), although LS mean point estimates are higher across the board following discontinuation, the confidence intervals do overlap, suggesting no significant change in sWASO after lemborexant discontinuation [4]. Consistent with this, Table 2 in our manuscript provides the same consensus recommendation for switching for all medications in the dual orexin antagonist class (a direct switch regardless of the class of medication switched to), including lemborexant.

In conclusion, this is an evidence-based clinical practice guideline. Our task was to provide useful advice for clinicians as they navigate the issue of deprescribing and switching medications. We reviewed the literature on insomnia medication deprescribing, tapering, and switching and rated the quality of evidence. We used this evidence to generate recommendations through discussion and consensus. No single study, and certainly no single figure in any single study, definitively guided our conclusions one way or another.
